# Contested Daily Routines, Contested Care. Children with Type 1 Diabetes in Covid-19 Times

**DOI:** 10.1007/s41255-021-00017-0

**Published:** 2021-04-14

**Authors:** Melike Şahinol, Gülşah Başkavak

**Affiliations:** Orient-Institut Istanbul (Research Field “Human, Medicine and Society), Istanbul, Turkey

**Keywords:** Children with Type 1 Diabetes (T1D), Continuous Glucose Monitoring (CGM), Covid-19, Socio-Bio-Technical Care Complex, Contested routines, Turkey

## Abstract

The conventional treatment of Type 1 Diabetes (T1D) is especially demanding for children, both physically and psychologically (Iversen et al. *Int J Qual Stud Health Well-being,* *13*(1), 1487758, 2018). Continuous Glucose Monitoring Systems (CGM) are an important aid for children and their families in dealing with the disease. In their work, however, Şahinol and Başkavak (2020) point out that CGM carry the risk of viewing T1D as a technologically solvable problem instead of considering the disease as a whole. This is mainly creating confidence in technology due to CGM experiences while neglecting significant dietary measures and exercises needed to be integrated into daily routines. During the current pandemic, this problem seems to take on a whole new level. Based on two periods of in-depth interviews and observations conducted with 8 families with T1D children aged 6 to 14 living in Istanbul and Ankara (Turkey) from May to November 2019 and again from May to June 2020, we compare and focus on the experiences prior to and during the pandemic time. We argue that despite the possibility of technological regulation of the disease, the vulnerability of children is increased and, more than ever, depends on socio-bio-technical entanglements.

## Introduction

In the twenty-first century, humanity has seen two major global pandemics with high levels of morbidity and mortality, both emerging from animal reservoirs: Severe Acute Respiratory Syndrome coronavirus (SARS-CoV) and Middle East Respiratory Syndrome coronavirus (MERS-CoV) (Paules et al., 2020). The current coronavirus disease, termed Covid-19 by the World Health Organization (WHO), has spread across the world rapidly (Del Rio & Malani, 2020). Coronavirus belongs to a large family of viruses being newly discovered. The current form is classified as 2019-nCoV and there are currently no clinically tested or proven vaccines or treatments (WHO, 2020a). On March 11, 2020, the WHO announced Covid-19 as a pandemic (WHO, 2020b). Meanwhile, overcrowded hospitals, overworked medical staff, rising death rates and the rapid, exponential spread of the virus have led to various political and legal interventions intended to handle the pandemic. Measures introduced include compulsory tests and travel bans, either compulsory or voluntary face masks, social distancing and either timed curfews or complete lockdowns of entire geographical areas. Rieck et al. (2020) point out that “major nodal points of such global flows, centres of commerce, travel and tourism like New York, Paris, London, but also Istanbul” are hit hardest by the pandemic but also different “cultural practices of coping and care” (ibid.). Coping with the pandemic differs in cultural and regional settings and further requires different approaches for different age groups. It, therefore, has a direct relevance to childhood care and development (Andresen et al., 2020). As we focus on families with T1D children during the pandemic in Turkey, we specifically elaborate on the coping practices adapted by these families in accordance with pandemic rules and regulations issued by the Turkish authorities before exploring the vulnerability of children in pandemic times.

Drawing on findings from our study on coping practices of families with T1D children, we examine the degree of relationship between the concepts of relevant childhood vulnerability, well-being and the socio-bio-technical care complex. By well-being we understand a rather broad concept, which is, however, considered here in direct connection with the handling of the children's disease and its care. For this purpose, we refer to the concept of Alexandrova (2017) who defines it as an “attempt to capture conditions that if realized in childhood enable children to grow up happier, healthier, and more positively connected to others” (101). We assume that these factors interact strongly with each other. At the same time, the vulnerability of children means to take into account their developmental needs, which has at its center the protection of children’s physical health (Raphael et al., 2006). After all, this is particularly at stake for vulnerable children with T1D during the pandemic. Vulnerability and care can both be conceived on different levels, and therefore, we must consider the family-specific coping practices in order to understand the vulnerability impact of Covid-19 pandemic measures on children with T1D.

## Background: Coping with the Pandemic in Turkey and Children with T1D in Covid-19 Times

Turkey seems to have responded quickly to Covid-19, even before it was declared a pandemic by the WHO. The distribution of free masks began at the end of January. Simultaneously, on January 24, 2020, airports introduced measures to detect and prevent the spread of the virus with infrared scans, the disinfection of all customs gates and the distribution of free masks and instruction leaflets. The first Covid-19 patient in Turkey was announced on March 11, 2020 (Anadolu Ajansı, 2020). On April 3, 2020, the Turkish Ministry of Health announced a temporary lockdown in order to prevent the spread of infections for the over-65 s and under-20 s (Evrensel, 2020). These strict quarantine conditions were challenging for families with children. While their parents could go outside, the children had to stay home. On May 13, 2020, after more than 5 weeks of quarantine, children under the age of 14 were allowed to go outside for four hours a day. On May 15, 2020, the regulations were changed to teenagers and young adults aged 15 to 20 now allowed outside four hours a day (BBC, 2020a). As of June 9, 2020, Turkish Prime Minister Erdoğan announced the end of restrictions for under 18-year-olds, provided they were accompanied by their parents or caretakers (BBC, 2020b). Before addressing the impact of these measures for children with T1D in Turkey, we provide an overview of current international research on diabetes and the ongoing pandemic.

A virus spreads across the world and has profound individual and societal consequences, such as on our health, our working and financial environment, our hygiene and travel behavior, and the way we treat our fellow human beings. Vulnerable groups of people are hit hard by these measures. Since the emergence of this virus, many medical studies have been published with special emphasis on the effect of chronic diseases (Clerkin et al., 2020; Henry & Lippi, 2020; Hussain et al., 2020; Saqib et al., 2020). The U.S. Centers for Disease Control and Prevention declares that people with chronic disease of Type 1 or Type 2 diabetes^1^ are a high-risk group for coronavirus (Dastmalchi, 2020). Numerous research articles and reviews focused on Type 1 and Type 2 diabetes have also been published. Some papers deal with the handling and management of diabetes during the pandemic (Hartmann-Boyce et al., 2020; Klonoff, 2020; Scott et al., 2020). These are mostly clinical studies aiming to “provide a better understanding of the coronavirus disease in people with diabetes, and its management” (Hussain et al., 2020). They focus on diabetes as a risk factor in the progression and prognosis of the coronavirus (Guo et al., 2020). Watts (2020) reports a “four times higher” risk of Covid-19 death rates for people with diabetes. Nevertheless, lockdowns seem to be elementary in the management of the disease (Bonora et al., 2020). Interestingly, the authors Bonora et al. (2020) report an improvement in glycemic control in patients with T1D during lockdown periods. The management of the disease in people who have stopped working due to the lockdown is particularly highlighted. They attribute this improvement to the fact that, despite limited opportunities for common physical activities (such as exercise), daily routines are slower, showing beneficial effects on T1D management. This is notable insofar as exercise is essential for regulating insulin levels. However, there is no evidence of the overall health determinants, such as the psychological and social well-being of these patients in their daily management of the disease during the pandemic period.

Parallel to the mentioned articles, a number of related scientific studies from various countries on Covid-19 with a focus on children with T1D were quickly published in medical journals. It is assumed that children who have a chronic illness are particularly affected and belong to the high-risk group for Covid-19: “Because ‘diabetes’ and chronically elevated hyperglycemia have been associated with worse outcomes in adults with Covid-19, the pediatric community has voiced great concerns about risk and outcomes for children with diabetes.” (DiMeglio et al., 2020) They focus on studies showing the link between Covid-19 infections and children with diabetes. Among them are those that claim there is no difference in the course the disease takes in children with or without diabetes. But DiMeglio et al. (2020) highlight that it

is important to remember that these articles only report data from children with type 1 diabetes. Given that obesity and hypertension are associated strongly with type 2 diabetes in youth and these comorbidities have been associated with more severe COVID-19 cases, children with type 2 diabetes may be at higher risk. (ibid.)

Some studies highlight the psychosocial impact of the pandemic on children and young adults with T1D without regular medical care or nutritional support in schools. In their analysis, Danne and Limbert focus on pediatric diabetology and draw attention to the rapid transition of health services to “remote care” and especially the issue of “the digital gap between pediatric and adult type 1 diabetes care” during the Covid-19 pandemic because “Covid-19 promote(s) autonomy of both young people and their parents in interpreting the data and making decisions” (Danne & Limbert, 2020a). A questionnaire-based cross-sectional study in Jordan, including 235 patients and families, analyzes the effect of strict quarantine conditions on pediatric patients with T1D in terms of deficiencies in insulin and glucose measurement resources. According to the findings, the lockdown measures caused a shortage in the supply of insulin and glucose measurement equipment for children with diabetes that was managed with classical diabetes methods (Odeh et al., 2020). Looking at the change in the incidence of pediatric T1D in Germany during the Covid-19 lockdown period between March and May 2020, Tittel et al. (2020) state that although “the incidence of type 1 diabetes could also have decreased, since people were less exposed to common infections”, however, a possible relationship between the Covid-19 pandemic and the incidence of T1D are still unclear (Tittel et al., 2020). Parallel to this research, Rabbone et al. (2020) find that there is a 23% reduction in new diabetes cases for pediatric T1D in 2020 compared to 2019 in Italy. A report from the UK highlights the increased numbers in new-onset T1D in children during the Covid-19 outbreak, and shows a contrary situation compared to Germany and Italy (Unsworth et al., 2020). D'Annunzio et al. (2020) analyze the effects of the Covid-19 infection on children and adolescents with T1D in Italy. According to their findings, infections are less severe in this diabetic age group than adult groups. Additionally, there is no evidence that the risk of coronavirus in children with diabetes is higher than in healthy children (d'Annunzio et al., 2020; Ng, 2020). However, “global epidemiological and clinical patterns of Covid‐19 among children with diabetes are still very limited” (Ng, 2020) so are social impacts and an understanding of the Covid-19 pandemic impact on the daily lives of children with T1D and, hence, their vulnerability. For this reason, we begin the next section with an introduction to the elementary concepts of vulnerability and diabetes technologies.

## Vulnerability, Diabetes Technologies and Care

Many determinants of a child’s development are already known, such as physical, emotional and cognitive development, but also moral and psychosocial development (Fernandez, 2014). A holistic understanding of children’s development is essential for an overall balanced development. But there are also other aspects such as cultural and geographical settings or economic conditions for instance (Andresen & Fegter, 2011; Andresen et al., 2012). Because of their immaturity and their dependence on others, such as parents, children are particularly vulnerable and need protection, care and help in their development (Finkelhor, 2008). Many determinants affect children’s well-being and vulnerability as they are interdependent. Andresen et al. (2012) indicate, that the

well-being of children in Germany is the granting of freedoms and the experiences of autonomy embedded among experiences of parental care within committed relationships. Hence, it is precisely a combination of freedom and parental care, of co-determination and protection that leads to a high life satisfaction in 6- to 11-year-olds. (Andresen et al., 2012, 437)

This seems particularly difficult in times of pandemics as various government regulations aimed towards protecting the population, the elderly and the chronically ill, not only lead to restrictions in freedom but also influences health in general – with consequences. There are very few medical studies pointing to a potential linkage between Covid-19 and new-onset T1D in children. Rabbone et al. (2020) point out the severe diabetic ketoacidosis (DKA) rates and new-onset T1D in children. Based on a study about children living in North-West London (UK) during the curfew from March 23, 2020, to June 4, 2020, researchers from Imperial College London state that

in comparison with a typical year, we estimate this represents an additional 12–15 new type 1 diabetes cases (80% increase) during the COVID-19 pandemic (…) We postulate that SARS-CoV-2 exposure contributed to the observed increase in cases by precipitating or accelerating type 1 diabetes onset.” (Unsworth et al., 2020, e1).

One of the reasons for this are also suboptimal lifestyle conditions (Gupta et al., 2020). In this context, it is important to point out, that any virus may cause blood sugars to be higher, so proper T1D management is essential. With pandemic regulations causing fewer daily activities the T1D management is at stake. Ghosh et al. (2020) emphasize the psychosocial impact of the pandemic and claim that children in general become vulnerable not through infection by the virus but rather psychologically due to quarantine, school closures, lack of physical activity or uncertainties about the epidemic. The authors highlight that children may develop anger, aggression and an overall disregard for society in the immediate future. All the more important is the motivation and love from their parents, especially in lock-down times. However, children with T1D using CGM are not only dependent on their parents as caretakers but also on (medical) technology to be provided. We emphasize that interdisciplinary childhood studies would benefit from a Science and Technology Studies (STS) perspective, taking into consideration the material surroundings for the well-being of vulnerable children. Concepts bringing together childhood and vulnerability are helpful in shedding light on systematic, structural and individual dimensions of childhood vulnerability (Andresen, 2014) but need to consider the meaning of materiality, like socio-technical structures and entanglements.

For T1D patients, including children who lack the essential insulin hormone in the body, the insulin pump (mostly combined with self-tracking devices^2^) allows them to keep their diabetes under “better” control as these devices monitor the glucose level and inject the necessary dose of insulin automatically. Compared to multiple daily insulin injections (MDI), CGM is a more comfortable alternative for patients, and would provide significant improvement in the hemoglobin A1c (HbA1c) levels in patients (Bergenstal et al., 2010; Litton et al., 2002; Pickup & Keen, 2002). Therefore, insulin pumps have positive effects on the health and well-being of T1D patients. In children, T1D is a particularly significant topic for public health services as this group is extremely vulnerable and might benefit significantly from new technologies (Wolkers et al., 2019).

Families of children diagnosed with T1D may experience anxiety in disease management. As the most classical diabetes management method, MDI, CSII or CGM can be considered the common option to handle children’s diabetes illness. As CGM devices have particular advantages and benefits such as glycemic control, the reduction of hypoglycemia or the decline in HbA1c, the usage of CGM is strongly preferred by parents (Litton et al., 2002; Pickup & Keen, 2002; Poolsup et al., 2013). As previously outlined in other studies, Şahinol and Başkavak (2021) show that CGM leads to a considerable comfort for the parents in their role as caregivers and for the children as care receivers since the need to be constantly pricked in the finger or woken up at night by their parents in order to measure insulin levels and receive insulin injections no longer applies. However, the authors present a more differentiated picture regarding the empowerment of care relationships. They explain that an imbalance between socio-bio-technical care practices leads to a back and forth of empowerment and disempowerment. In their concept of “socio-bio-technical care complex”, Şahinol and Başkavak (2021) point out that a holistic care approach is essential for digital self-tracking technology such as CGM to empower the patient, and especially children. In body care such as exercise and diet control, it is observed that a form of self-control negligence can occur with the use of technology. After all, the control of insulin levels is delegated to technology, and this has implications for the entire care cycle. The authors state that while adults can keep themselves relatively more in control of diet and are aware of their bodies’ need to exercise it has been observed that children experience disempowerment in this situation. As caregiving parents stated throughout the interviews, one of the biggest sources of stress for their children was the impaired metabolic control and weight gain as they felt comfortable in eating how much and whatever they wanted. The authors conclude that CGM carry the risk of viewing T1D as a technologically solvable problem instead of considering the disease as a whole. This is mainly due to the CGM experiences creating confidence in technology while neglecting important dietary measures and exercises needed to be integrated into daily routines.

As the global pandemic conditions have forced restrictions on families and their children with T1D in various ways, diabetes management and experience have changed as a result of staying at home and indoors for many hours straight decreasing daily physical activity. Daily physical activity is not only important in regulating insulin levels. We argue that during the pandemic period while considering T1D as a technical solvable problem without paying sufficient attention to holistic care, the vulnerability of children living with this health condition can skyrocket. We conceptualize our thoughts at the interface of body and technology and describe these processes in the light of posthuman cyborg theories. The latter especially point out the materiality within organic–inorganic entanglements (Braidotti, 2019; Haraway, 1995; Şahinol, 2016, 2018). Oudshoorn (2016, 2020), who addresses a particular vulnerability that results from the fact that medical technology enters deep under the skin, describes “how defibrillators introduce two new kinds of vulnerabilities: vulnerability as an internal rather than an external threat, and as harm you may try to anticipate but can never escape” (Oudshoorn, 2016). So, if we look at vulnerabilities that affect the distribution of actions between humans and machines or technologies, we must also include their materiality in the analysis. A theory of vulnerability in childhood must, therefore, account for the power relations that are renegotiated by materiality.

Next, we show if and how daily routines and challenges for children with T1D and CGM change and discuss impacts on their vulnerabilities.

## Methodology

Based on two periods of in-depth interviews and observations conducted with 8 families with T1D children aged 6 to 14 living in Istanbul and Ankara (Turkey) from May to November 2019 and again from May to June 2020, we compare and focus on the experiences prior to and during the pandemic time. The participants who were recruited for the 2019 interviews, were connected again remotely between May 14 and September 1, 2020. Severe quarantine conditions were in place at the time. To be included in the study, parents with a T1D child had to be using a CGM device for at least 1 year prior to the interview. We connected with the parents of T1D children via instant messages or phone to invite them for participation in the follow-up study. The interview questions were circulated via e-mails. Two-round e-interviews were conducted with 8 parents whose children (3 female, 5 male) with T1D ranged in age from 6 to 14 years. Interviewees were mostly from Istanbul and Ankara.

To ensure anonymity, participant names are converted into name codes such as UAuser26 for example. The two initial letters are random selections from participant names and surnames followed by the relevant abbreviation according to the actor group they belong to, followed by a number, which is in order of an interview set of elements. As this is a follow-up study focusing on interviews with users (here the parents) of CGM one will only find the ‘user’ abbreviation.

The qualitative data was jointly analyzed by drawing on coding, categorizing and conceptualizing methods pertaining to grounded theory (Strauss & Corbin, 1996) using the qualitative data analysis software ATLAS.ti (v. 8.4.24). Paradigm model, field notes and memos were shared on a weekly basis and preliminary research results were discussed on a bi-weekly basis, allowing for permanent comparisons, refinements of research questions and joint formulations of hypotheses.

## Findings

Our daily life and behavior are framed within a specific structure in a specific society. But in a world surrounded by technology, technology is also shaping our daily lives and, therefore, becomes a dimension to everyday social interaction. This is also the case for chronically ill children equipped with medical technology like CGM. Techno-bodily experiences related to health or care and the evidence of their intra- and interactive dynamics in interactions have been discussed from posthuman and cyborg perspectives and/or in disability studies (Kafer, 2013; Oudshoorn, 2016, 2020; Şahinol, 2016, 2018; Selinger, 2008; Spreen, 2010). In the following section we illustrate how materiality is part of childhood vulnerability addressing the impact of the pandemic measures on children with T1D in Turkey.

## Structural and Techno-Medical Impacts on Children with T1D using CGM

As mentioned previously, children had to face a 5-week curfew until May 13, 2020 followed by less restrictive lockdown rules until mid-June 2020. With the pandemic new health regulations were enforced in Turkey. Before discussing the impact of the curfew on the daily lives of families with T1D children, we must analyze the impact of regulations in medical care in combination with technology, and especially CGM, on these families.

Children diagnosed with T1D need lifelong treatment once the disease occurs. As many parents revealed in our interviews, being a parent of a child with T1DM means dealing with big challenges, such as bureaucratic barriers or economic difficulties. It has been observed that children with T1D and their parents were very eager to use the CGM due to enabling better blood glucose control. Access to such technologies is difficult because both, the public and private health system contributions, are significantly limited in Turkey. Parents stated that they were straining their economic conditions to be able to afford such a device. They take out loans or sell their cars for example. Despite challenging financial conditions, they try buying these devices with the intention to better facilitate the management of the disease. The role of the private health sector in diabetes technology is important as our interviewees state. Especially families with T1D children experience difficulty in receiving the needed financial support from the Turkish health care system because CGMs are not fully covered by the Social Security Insurance (SSI). The price of insulin pumps has risen by almost twice the previous price despite low wage increases and high inflation rates in Turkey in the last years. The monthly costs of necessary medical supplies increased at that level, too, making it almost equal to the minimum wage. The amount refunded by the SSI remained the same despite the high increase in prices. Many interviewees criticized that the Turkish healthcare system only pays a very small portion of the costs of insulin pumps. According to the announcement of the Turkish Social Security Institution dated 1.4.2020 (Social Security Institution, 2020), a new regulation was put in place during the Covid-19 period due to the possibility of an infectious disease occurring more severely in people whose immune system is adversely affected due to chronic disease. According to this new regulation, people with valid medical reports registered in the e-health system could pick up their medicines directly from the pharmacy without the need of a prescription from a doctor or a hospital. Every parent stressed the importance of CMG for their lives but also of the new regulations with regards to prescription procedures during the pandemic (i.e. MRuser28). The crisis transformed the health care system outwardly to the benefit of the families who can receive medical supply in an unbureaucratic, timely and, thus, safe manner.

The drug supply system, which we have been asking for and demanding for years but has never been changed and instead turned into a torment for us, has become easier! During the pandemic period, we can access consumables such as insulin strips and pumps without a prescription. We hope that this system will always remain like that. (UAuser26)

Another interviewee holds a similar view by stating that the “procurement was easier during this period as the medication reporting periods were extended from the insurance system and the obligation for the print prescriptions for chronic diseases such as diabetes was removed.” (SEuser24).

Because the purchase of medical supplies is processed using the Turkish National ID to which health data is also linked (unlike in Germany) medical errands were easier to run. This example clearly shows the importance of digitalizing the healthcare system.

We accessed both consumables, insulin and glucometer strips more easily than before because there was no prescription requirement. We bought all our stuff from related places on a monthly basis with the ID number. In fact, we understood how report and prescription procedures for chronic diseases is unnecessary, that a more convenient procedure could easily be established based on reports only. (LSuser17)

The healthcare infrastructure is, therefore, an important “material” factor as it has a structuring effect on the well-being of patients and their families. The *socio-technical* management of health care not only has a calming and thereby positive effect on the condition of families and children, it is also appropriate to the vulnerability of the children with T1D. However, Kayaalp and Isik (2020) suspect that Turkey's success in fighting the pandemic is due to infrastructural deficiencies and, among other things, an intentional lack of regulations. They also underline the resulting flexibility of the system, which has made it possible for the Turkish government to react quickly. Therefore, one can draw the conclusion that the more inflexible a techno-medical system is, and with it state-regulated technical access to medical materials, the more vulnerable the chronically ill child who depends on these materials.

## Explaining the Pandemic to Children and Taking Measures

We asked all parents how they explained the Covid-19 situation to their chronically ill child(ren). Since these children belong to the risk group due to their chronic disease, we expected them to communicate to the children about this particular risk. Although all families expressed their special concern for their children with T1D, because of their “low immune system” (EZuser27) for instance, they did not talk to their children about it. Only one parent said that his children (one of them with T1D) “improved their knowledge of protection against infectious diseases a little more during this outbreak than previously” (SEuser24). Interestingly, most parents pointed out that they felt the situation was a state of emergency as usual. To them life with the disease is already a permanent state of emergency. The following statement by a father is remarkable in that respect. His two children aged 8 and 13 were both diagnosed with T1D about 7 years ago. He draws an analogy between T1D and the pandemic saying that families with T1D children have more of an advantage as compared to families with healthy children because they are already trained for emergency situations: “Homes where children with T1D live are already managing a pandemic throughout their lives. In this sense, we are somewhat luckier than other families, or, correctly, ‘trained’. I think it's a chance to even keep an out-of-touch diabetic in front of us.” (LSuser17).

While talking to their T1D children about the pandemic, it is likely that their explanations may vary and reflect the level of personal anxiety due to the illness. However, such differences were not observed in the field. The ages of the children participating in the study ranged between 6 to 14 years. The parents pointed out that they explained this new global pandemic and the resulting quarantine measures to their T1D children the same way they did to their healthy children: “My child is 14 and we didn't need to come up with a special explanation. There is enough information flow on social media and television. She followed the agenda as much as we did.” (NAuser15) The mother with the youngest T1D child (6 y/o) said that the parents explained the coronavirus to him as such:

We sat down and explained it very clearly so that he could understand. He took it quite normal. We explained the rules he would need to know. Then he studied it on the internet and watched about it. He was telling everyone that this was a mutated virus. My son was not affected much. (MRuser28)

One father with two sons aged 6 and 13 (T1D) said that he did not treat his T1D child differently from his healthy child:

First of all, as parents we did not panic about the virus. The children improved their knowledge of protection against infectious diseases a little more than before. While emphasizing the necessity of washing hands and wearing a mask outside we also took maximum care. We tried to develop a sense of responsibility towards others emphasizing the awareness that the negative effects of the virus mostly harm elderly people. (SEuser24)

Parents with two children suffering from T1D stressed that they were rather pragmatic about explaining the epidemic and precautions. They were strict about the rules and, after a while, their children adapted well to these “new normal” conditions:

Frankly, as much as we were panicking, we chose to put them on high alert at the same time. As a result, we did not downplay a disease with which millions of people were infected and that caused hundreds of thousands of people to die within 6 months just so that the psychological well-being of our children would not decline. We completely cut off their contact with the outside world. We followed the hygiene rules in an extremely strict manner. Interestingly, after a while, we observed that they got used to this new way of life and started to live without fear as compared to before yet remaining cautious. Even after curfew was lifted, we make sure that they do not insist on going out. (ASuser17)

All parents did say that they increased precautions, hygiene measures and reduced physical affection significantly. One statement is exemplary:

thought of my son more than myself. Of course, I was worried that he is more at risk because he has diabetes. We certainly showered immediately when we went out and back home in order not to infect him. We disinfected ourselves, avoided close contact with my son, we didn’t kiss or hug him. (BCuser14)

## Technology and Well-Being of Children with T1D during Covid-19

Both, the increase in the quality of life and the physical well-being during the daily routine of the disease, have been expressed by the participants many times. Transitioning to CGM implies “fewer finger punctures and less pain” (LSeng18) for most participants as reported in the first and, also, in the follow-up interviews. Some parents even stated that average insulin levels decreased during the pandemic period. Parents rated this as positive and attributed this to insulin regulation by means of the device:

CGM becomes vital, especially in times of such radical life changes. (…) Before the pandemic period, our HbA1c measurements were 6.5 for my daughter and 6.1 for my son. During the pandemic period (2 months), our measurements are 5.9 and 5.4. We have closely witnessed the positive results of the interactive and more effective use of diabetes technologies. In summary, we have turned the concern that diabetic patients are one of the patient groups most severely affected by the epidemic into an advantage thanks to technology. Moreover, with a daily life as if ‘there is no diabetes’. (LSuser17)

However, this positive impact of technology must be viewed in direct relation to the presence of the parents as caregivers and their controlled efforts, including the structuring of daily routines:

The pump has a lot of benefits here. Being able to directly interfere with the pump, insulin makes life easier for us mothers and fathers. Having much time at home, life is a little more flexible. Waking up late in the morning and having a late breakfast becomes more controllable with the ease of the pump. (UAuser26)

This is mainly due to parents being able to check almost countless measurements with the device. The parent in the below section stated and drew attention to an important fact that changed during the pandemic: The technology helped to regulate the insulin level according to the new daily routine. The fast, almost automatic regulation combined with control was crucial:

The insulin pump is a technology to manage exactly such extraordinary situations in terms of design and method of treatment. An extraordinary period/situation actually covers every period when habits and activities change in diabetes management. (…) Actually, the pandemic is just one of these situations. In other words, we can call any period that requires changes and regulation to insulin doses as extraordinary periods. Technology saves time and provides flexibility when making these changes. (…) A diabetic who takes finger measurements can make a maximum of 24 measurements per day, even if they measure every hour, whereas a diabetic person using CGM can see 288 measurements per day. (ASuser19)

At the same time, it was underlined that the curfew meant that parents could constantly monitor the child’s insulin level with the device. On the one hand, this possibility and empowerment of control meant that the parents “could take this process as an opportunity to get to know [their] child and [it’s] diabetes better.” (LSuser17) On the other hand, however, the constant control option was problematic at times, as different types of anxiety developed because of increasing insulin levels or due to T1D children belonging to the high-risk groups for instance:

It affects us as much as it affects families without children with diabetes. Of course, I can say this with confidence because not being exposed to any complications of diabetes and the confidence of good diabetes management. Otherwise, you know that complicated diabetes management is very risky due to a diabetes pandemic with poor management. Therefore, it is inevitable to have an anxious and tense period. (SEuser24)

Continuous and simultaneous data availability might lead to constant and excessive control of the health status causing compulsive control behavior or leading to regulations by digital health tracking (Şahinol & Başkavak, 2020). The possibility of constantly monitoring the child’s insulin levels together with the parents' fear, as addressed in many interviews, was observed beyond the mere daily control routines. Due to the constant presence of numbers the eating habit of the child is permanently monitored and controlled:

We are those who experienced the epidemic from the positive side... Because it enabled us to minimize hyperglycemia and hypoglycemia in terms of monitoring my son's diabetes since we were at home (…) Normally, it was a little difficult to keep diabetes under control for the whole day due to school and work. Nowadays, we have taken better control because we were all at home. (…) To compare, when I am not there, my son eats cheat meals (chocolate, ice cream, candy, crackers, dried nuts, etc.); when I'm with him, my son eats fewer of these and since I constantly control the pump, I can detect these cheat meals and send insulin immediately. (BCuser14)

Eating habits and dietary measures are important for children with T1D. “At the same time, being at home constantly changes our eating habits. It leads to more consumption.” (SEuser24).

This situation in controlling the child’s behavior influences not only a healthy insulin level but the well-being of the child in a contrary manner. A mother explained the interconnections this way: “Of course, this process stressed us out. Because my son is only 6 years old. It was difficult for me to entertain him. After a while, I could not even give him enough as a mother. He became aggressive and changed his temper.” (MRuser28) Another parent reported anxiety and sleepless nights and their decreasing patience for the child. “Our sleep disorders (as parents) increased since the end of April, when the epidemic peaked. Our tolerance and patience for the wishes of the children decreased. During this period, we burnt out psychologically.” (LSuser17) Nevertheless, the parents’ and their children’s psychological states are important for the overall well-being of the children. Increasing stress levels effect the child’s well-being as explained by parent MRuser28: “My son had too much stress. We saw the blood sugar level of 400–450 once or twice.” (MRuser28) MRuser28 refers to the changed daily routines and, thus, the *socio-technical structure* as a stress factor due to the curfew and the pandemic. In the next section, we show how mentioned changed daily routines affect the children’s well-being.

## Contested Daily Routines and T1D Children

The constant monitoring of insulin levels may be demanding at times. It, nevertheless, provides crucial assistance for the parents and their children. By checking the levels, one can adjust the therapy schedule, such as regulated mealtimes and exercise. The parents, however, all complained that their order had been disrupted, that social isolation and the constant crowding out encouraged arbitrary eating habits and reduced the motivation for activities at home. “Reduced exercise, changed routines, and increased food intake are frequent reasons for adapting the therapeutic regimen” (Danne & Limbert, 2020b). EZuser27 explained the connection between inactivity at home during the curfew and the increasing insulin level despite having regular meals: “Although, we didn’t make big changes to our meal order, inactivity at home caused some increase in his [son’s] insulin usage.” (EZuser27) Another parent attributed this to the fact that the child's stress level increased due to changed routines and irregularity:

And the most important thing is that our mealtimes are messed up because our order is over. He [the son] slept very late at night and woke up late in the morning and all his meals times were upset. (…) I didn't get support because I know how to correct the dosage. I sent the amount of insulin as needed, and then it returned to normal. (MRuser28)

Almost all parents increased the insulin dosage, as “Inactivity causes sugar to rise on its own. We deal with this by increasing by 20% as the easiest remedy dose.” (UAuser26) But insulin injections bear risks:

We experienced difficulties especially in terms of physical activity (exercise) due to curfews. Before the pandemic, insulin doses were specially adjusted according to the post-meal activity (breathing time, playing outside, etc.). In addition, we used to reduce it with exercise during periods of high sugar. During the pandemic period, it was not possible to convince the child to exercise after meals and to design the physical conditions of the house. This led us to producing extra insulin that we could reduce with exercise from time to time. This is a cause for concern, considering that every extra dose of insulin increases the risk of hypoglycemia. (ASuser19)

We can see that considering T1D as technical solvable problem might affect the well-being and, thus, the vulnerability of children with T1D. We know from other parents, that “sports and physical activity are important for every individual, even more important for those with Type 1 Diabetes.” (SEuser24) Nevertheless, the decreasing motivations during the pandemic seemed to be an intervening condition (e.g. ASuser19), even when parents were aware of the need for a holistic care. Regular physical activity and exercise are essential to controlling blood sugar levels in T1D. Several studies stress that physical activity is directly related to reduced insulin requirements (Chimen et al., 2012). As it becomes clear from the interviews, the continuous well-being of the child is only maintained in combination *with bio-technical regulation and the socio-technical settings*, such as doing sports at home via YouTube videos for example. Personal motivation during curfew seemed an important factor: “I can say that the level of physical activity dropped to zero. We were having good times at first. We were doing exercises with him at home. But at the end, he got bored of it too. And I couldn't make him do anything.” (MRuser28).

There were, however, some families who could include exercises in the daily program and motivate their children with a variety of possibilities like playing in the garden (EZuser27), watching exercise videos on YouTube or doing sports using apps. It seemed that a variety of technically mediated possible activities increased the motivation:

Since we do her exercises regularly at home and have the opportunity to use the garden of the house, we obviously did not see any negative effects. During the quarantine days when we were locked down at home, my daughter did a daily exercise program (around 30-60 minutes) with the help of some apps and videos. When we went out to the garden, we took daily 1-hour walks together. During this time, we did not observe weight gain or blood sugar deterioration due to inactivity. (NAuser15)

It seems, however, that despite technical resources such as apps and videos, social isolation led to the same boring, monotone structures demotivating children over time:

We tried to pay attention because a sedentary life is unhealthy and leads to weight gain ... We played ball at home, played sports with videos on the internet, tried to increase physical activity, but still it takes a short time to exhaust all possibilities inside the house and it causes monotony for the child after a while. (BCuser14)

When parents could not motivate their children to exercise at home, they tried to reduce insulin levels by adding insulin. This, in turn, carried the risk of the child developing hypoglycemia causing a direct impact on the child's well-being.

As is known in diabetes, physical activity and sports have an important place. We know that physical activity and sports create an insulin effect. During this period, there was a limited exercise area because we had to stay at home. We were able to compensate for this by increasing our basal insulins a little to make our blood sugar more regular. (SEuser24)

Being bored affected children’s school performance in some cases. BCuser14 said: “My son was not exactly an active, sports-loving child since his childhood. (…) Being at home didn't affect him too much. Even he was very happy at the beginning, but later on he began to feel bored and missed his school.” (BCuser14).

The above examples show that although socio-technical entanglements cause bio-technical adjustments (insulin regulation by technology) motivation in children seems an additional key element in implementing daily routines for children with T1D. It underlines the importance of a holistic care for all relevant components found in socio-bio-technical care complex (Şahinol & Başkavak, 2021).

## Discussion

This research is the first to investigate the revised daily care and illness routine experienced by parents with T1D children during the Covid-19 lockdown period in Turkey. Our results correspond with Bonora et al. (2020) who report an improvement in glycemic control in patients with T1D during lockdown. The improvement was not related to limited opportunities to activities but to the availability of and access to data for parents due to crowding. We show important consequences for the psychological well-being of parents as caregivers and their T1D children as care receivers caused by the lockdown. These factors intervene with technical structures and socio-technical entanglements. In accordance with Ghosh et al. (2020) who pointed to the psychosocial impacts of the pandemic for children, we were able to outline a broader picture by relativizing the impacts due to quarantine or lack of physical activity. In our study, we emphasize that children with T1D become vulnerable by not managing the disease as a whole during pandemic times. The findings show that many families increased their insulin dosage some by as much as 20%. This was often the case when parents were either unable to motivate children to exercise and pay attention to their diet or when children were generally more stressed, or parents viewed technology as the solution to the problem. The simultaneous control mania of parents led to reactions between parent(s) and child(ren). Consequently, this creates a special vulnerability for the not only the chronically ill child, but also for the child-parent relationship. Thus, the child-parent relationship during the pandemic was not our main focus. The implications of these findings suggest a closer examination of everyday scenarios that are affected by the parent–child relationship during the pandemic. However, this relationship is considered as to be embedded in the care complex therefore does not remain unaffected as elaborated above.

In addition, despite the variety of physical and mental entertainment provided by the parents to the children, most child(ren) became demotivated over time through a normalization effect. This shows how psychosocial factors remain an essential key in coping with the challenges of the pandemic, although bio-technical adjustments, technical aids and socio-technical infrastructures are also essential. We conclude that the more inflexible a techno-medical system is, and with it state-regulated technical access to medical materials, the more vulnerable a chronically ill child depending on these materials becomes. We highlight, that several materialities must be embedded in psychosocially supportive socio-technical settings as the vulnerability of T1D children using CGM is increased by large proportions and, more than ever, depends on socio-bio-technical entanglements. Therefore, we include the importance of various facets of materiality into the analysis of childhood vulnerabilities in Covid-19 times. In doing so, we show how vulnerabilities of children with T1D and their families coped within distributed actions between humans and machines/technical aids with the pandemic in their daily routines.

## Footnotes

1 Diabetes is considered a severe chronic illness when the pancreas stops producing enough insulin (WHO, 2016).

2 Continuous Glucose Monitoring (CGM) is a general concept including various kinds of devices. As proposed by (Allen & Gupta, 2019), these devices can be categorized into two groups: “standalone” devices and “CGM integrated with insulin pumps” or “continuous subcutaneous insulin infusion (CSII)” The main difference between these two groups is the use of insulin pumps in the latter, while devices in the first category only track the blood glucose level and the patients must continue to inject insulin themselves. In our study, all participants except one use “CGM integrated with insulin pumps” usually referring to the device as “insulin pump”.
